# The efficacy and safety of plasma exchange in patients with sepsis and septic shock: a systematic review and meta-analysis

**DOI:** 10.1186/s13054-014-0699-2

**Published:** 2014-12-20

**Authors:** Emily Rimmer, Brett L Houston, Anand Kumar, Ahmed M Abou-Setta, Carol Friesen, John C Marshall, Gail Rock, Alexis F Turgeon, Deborah J Cook, Donald S Houston, Ryan Zarychanski

**Affiliations:** Department of Internal Medicine, University of Manitoba, GC425-820 Sherbrook Street, HSC, Winnipeg, R3A 1R9 Canada; Department of Haematology and Medical Oncology, CancerCare Manitoba, 675 McDermot Ave, Winnipeg, R3E 0V9 Canada; Faculty of Medicine, University of Manitoba, 250 Brodie Centre, 727 McDermot Ave, Winnipeg, R3E 3P5 Canada; George & Fay Yee Center for Healthcare Innovation, University of Manitoba/Winnipeg Regional Health Authority, GE706-820 Sherbrook Street, HSC, Winnipeg, R3A 1R9 Canada; Neil John Maclean Health Sciences Library, University of Manitoba, Brodie Centre, 727 McDermot Ave, Winnipeg, R3E 3P5 Canada; Section of Critical Care Medicine, St. Michael’s Hospital, 30 Bond Street, Toronto, M5B 1W8 Canada; Ottawa Hospital, 501 Smyth Road, Ottawa, K1H 8L6 Canada; Division of Critical Care Medicine, Department of Anesthesiology and Critical Care Medicine and Population Health and Optimal Health Practices Unit, CHU de Québec Research Center, Université Laval, 1401-18th Street, Québec, G1J 1Z4 Canada; Department of Medicine, McMaster University, Hamilton, L8S 4K1 Canada; Department of Clinical Epidemiology and Biostatistics, McMaster University, 1280 Main Street, West, Hamilton, Canada

## Abstract

**Introduction:**

Sepsis and septic shock are leading causes of intensive care unit (ICU) mortality. They are characterized by excessive inflammation, upregulation of procoagulant proteins and depletion of natural anticoagulants. Plasma exchange has the potential to improve survival in sepsis by removing inflammatory cytokines and restoring deficient plasma proteins. The objective of this study is to evaluate the efficacy and safety of plasma exchange in patients with sepsis.

**Methods:**

We searched MEDLINE, EMBASE, CENTRAL, Scopus, reference lists of relevant articles, and grey literature for relevant citations. We included randomized controlled trials comparing plasma exchange or plasma filtration with usual care in critically ill patients with sepsis or septic shock. Two reviewers independently identified trials, extracted trial-level data and performed risk of bias assessments using the Cochrane Risk of Bias tool. The primary outcome was all-cause mortality reported at longest follow-up. Meta-analysis was performed using a random-effects model.

**Results:**

Of 1,957 records identified, we included four unique trials enrolling a total of 194 patients (one enrolling adults only, two enrolling children only, one enrolling adults and children). The mean age of adult patients ranged from 38 to 53 years (n = 128) and the mean age of children ranged from 0.9 to 18 years (n = 66). All trials were at unclear to high risk of bias. The use of plasma exchange was not associated with a significant reduction in all-cause mortality (risk ratio (RR) 0.83, 95% confidence interval (CI) 0.45 to 1.52, I^2^ 60%). In adults, plasma exchange was associated with reduced mortality (RR 0.63, 95% CI 0.42 to 0.96; I^2^ 0%), but was not in children (RR 0.96, 95% CI 0.28 to 3.38; I^2^ 60%). None of the trials reported ICU or hospital lengths of stay. Only one trial reported adverse events associated with plasma exchange including six episodes of hypotension and one allergic reaction to fresh frozen plasma.

**Conclusions:**

Insufficient evidence exists to recommend plasma exchange as an adjunctive therapy for patients with sepsis or septic shock. Rigorous randomized controlled trials evaluating clinically relevant patient-centered outcomes are required to evaluate the impact of plasma exchange in this condition.

**Electronic supplementary material:**

The online version of this article (doi:10.1186/s13054-014-0699-2) contains supplementary material, which is available to authorized users.

## Introduction

Severe sepsis and septic shock are among the leading causes of death in patients admitted to an ICU worldwide and, despite advances in treatment and supportive care, the mortality remains greater than 20% [1-4].

Plasma exchange or plasma filtration involves the separation of plasma from whole blood, removal of the plasma, and replacement with normal saline, albumin, or fresh frozen plasma [5,6]. Plasma exchange has the potential to improve survival in sepsis by restoring homeostasis through the removal of harmful substances (for example, bacterial toxins, activated complement and coagulation factors and inflammatory cytokines) and, when the replacement fluid is plasma, replacement of deficient blood components (for example, coagulation factors and natural anticoagulants) [7]*.* Plasma exchange, however, also has the potential to cause harm by diluting or attenuating the host’s adaptive response to infection.

Case reports and small observational studies in humans with septicemia or meningococcemia suggest a survival benefit of plasma or whole blood exchange when compared with expected survival rates [8-16]. While some studies have reported a fourfold increase in survival compared with historical controls [16], others have found relative survival benefits of 4 to 25% [13,15]. The small sample size of these studies and the lack of a contemporaneous comparator limit our ability to draw conclusions regarding the risks and benefits of plasma exchange in sepsis and septic shock. Currently, the American Society for Apheresis lists plasma exchange as an experimental treatment for sepsis and multiorgan failure, and considerable practice variability exists in the application of apheresis technologies for the treatment of sepsis [6]. The objective of this systematic review was to examine the efficacy and safety of plasma exchange compared with usual care in patients with sepsis or septic shock.

## Methods

Using an *a priori* published protocol (CRD 42013004290), we conducted our systematic review in accordance with the Methodological Expectations of Cochrane Intervention Reviews guidelines [17] and reported our results as per the Preferred Reporting Items for Systematic Reviews and Meta-analysis guidelines for systematic review and meta-analysis [18]. A review team comprised of experts from multiple fields (hematology, critical care, research methodology and library sciences) formulated the research question, reviewed the search strategy and review methods, and provided input throughout the review process.

### Research question

Our primary research question was: ’In critically ill patients with sepsis, severe sepsis, septic shock, or disseminated intravascular coagulation due to infection, is plasma exchange, compared with usual care, associated with differences in mortality, ICU and hospital length of stay, and central venous catheter-related complications?’ (Additional file 1). We included trials that met the following criteria: prospective randomized trials of human subjects; trials enrolling adults or children; at least 80% of patients diagnosed with sepsis, severe sepsis, septic shock or disseminated intravascular coagulation due to infection; and plasma exchange or plasma filtration (regardless of timing, number of treatments, replacement fluid or frequency of administration) was compared with placebo or usual care. Conventional dialysis or hemofiltration were not considered as plasma exchange, because the modalities differ in terms of plasma protein removal and replacement fluid. Our primary outcome was all-cause mortality reported at longest follow-up. Secondary outcomes included ICU and hospital lengths of stay, and safety outcomes included central venous catheter-related complications (infection, thrombosis) or procedural related complications. Additional file 2 presents inclusion and exclusion criteria.

### Search strategy and study selection

We searched the following electronic databases from inception to 28 April 2014: MEDLINE (PubMed), EMBASE (Ovid), and CENTRAL (the Cochrane Library – Wiley). The Cochrane Highly Sensitive Search Strategy was used as a model for searching [19]. The original search strategy was designed for MEDLINE with input from an information specialist and then translated for other databases. The following search terms were used: ‘sepsis; shock, septic; disseminated intravascular coagulation; thrombocytopenia; plasma exchange; plasma filtration; and blood component removal’ (Additional file 3). We performed forward searches of included trials and relevant reviews in Scopus to identify additional citations. To identify ongoing or planned trials, we searched the World Health Organization’s International Clinical Trials Registry Platform. In addition to electronic database searching, we searched abstracts and conference proceedings for the following societies from 2008 to 2012: American Society of Hematology, European Hematology Association, American Thoracic Society, International Symposium on Intensive Care and Emergency Medicine, Society of Critical Care Medicine, European Society of Intensive Care Medicine, and American Society for Apheresis. Finally, we hand-searched the bibliographies of relevant reviews and included trials for additional citations. Reference management was performed in EndNote™ (version X6; Thomson Reuters, Philadelphia, PA, USA). Two reviewers (ER and BLH) independently reviewed the title and abstract of each citation to determine whether a study generally met the inclusion criteria. The full text of all citations listed as ‘include’ or ‘unsure’ by either reviewer at this stage of screening were retrieved for full-text review. The full-text versions of potentially relevant citations were then independently assessed to determine whether the trial satisfied the inclusion and exclusion criteria. Discrepancies between reviewers were resolved through consensus in discussion with a third reviewer (RZ).

### Data extraction and management

Two reviewers (ER and BLH) independently extracted data from included trials using standardized and piloted data extraction forms. Discrepancies between reviewers were resolved through consensus in discussion with a third reviewer (RZ). We extracted data including trial demographics (author, year of publication, country, funding source, publication status, duration of follow-up, number of centers involved, inclusion and exclusion criteria, and methodological quality using the Cochrane Collaboration Risk of Bias tool) [20,21]. In addition, we extracted patient demographics (age, sex, ICU admission diagnosis), Acute Physiology and Chronic Health Evaluation II score [22] or other illness severity score, number of patients requiring mechanical ventilation or vasopressors at baseline, co-interventions in the ICU (antibiotic use, vasopressors, ventilator, corticosteroids, renal replacement therapy), details of the intervention (type of apheresis procedure, number of treatments administered, volume exchanged, replacement fluid used) and details pertaining to the primary, secondary, and safety outcomes. Data management was performed using Microsoft Excel 2011 (Microsoft Excel for Mac 2011. Microsoft Corp. Redmond, WA, USA).

### Quality assessment

We used the Cochrane Collaboration Risk of Bias tool to assess the internal validity of included trials [20,21]. This tool consists of six domains (sequence generation, allocation concealment, blinding, incomplete outcome data, selective outcome reporting, and other sources of bias) and a categorization of the overall risk of bias. Each separate domain is rated low risk, unclear risk, or high risk. If one or more individual domains were assessed as having a high risk of bias, the overall score was rated as having a high risk of bias. The overall risk of bias was considered low only if all components were rated as having a low risk of bias.

### Data analysis

We analyzed data from the included trials using Cochrane Review Manager (RevMan version 5.1, 2011; The Cochrane Collaboration, Copenhagen, Denmark). For dichotomous data, we expressed summary measures of effect as risk ratios (RRs) with 95% confidence intervals (CIs) using the Mantel–Haenszel method. We used a random-effects model for all analyses. A RR of less than 1 suggests a lower rate of death among patients treated with plasma exchange than those in the control group. We assessed statistical heterogeneity of the data using the *I*^2^ statistic. We investigated potential sources of heterogeneity using subgroup analyses based on methodological characteristics, quality and patient characteristics. Due to the small number of included trials, systematic evaluation of publication bias was not possible.

### Subgroup analyses

To investigate potential statistical heterogeneity, we performed subgroup analyses in several prespecified groups, including children versus adults and trials conducted in North America versus in other continents.

## Results

**Figure 1:**
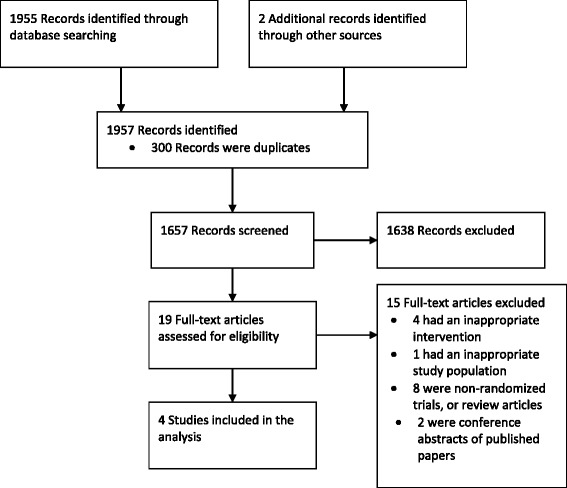
**Preferred Reporting Items for Systematic Reviews and Meta-analysis study flow diagram.**

Of the 1,957 records identified from electronic and hand searches, we included four unique randomized trials enrolling a total of 194 patients (Figure 1, Table 1) [23-26]. All were published in peer-reviewed, English-language journals. All trials were at unclear to high risk of bias (Table 2). One trial was conducted in North America [25], while the other three were conducted in Europe [24], Australia [23] or both [26]. Two were multicenter trials [23,26]. One trial enrolled adults only [24], one trial enrolled both adults and children [23] and two trials enrolled children only [25,26]. The mean age of adult patients ranged from 38 to 53 years (*n* = 128) and the mean age of children ranged from 0.9 to 18 years (*n* = 66). The mean Acute Physiology and Chronic Health Evaluation scores were 25.2 (APACHE II) [23] and 54.9 (APACHE III) [24] in the two trials reporting baseline severity of illness. Details of the intervention varied considerably among the included trials (Table 1). The interventions ranged from a single plasma exchange treatment [24], to 34 to 36 hours of continuous plasma filtration [23,26], to daily plasma exchanges for 14 days [25]. Co-interventions were poorly reported, with two trials reporting the number of patients requiring ventilator and vasopressor support [24,26].

**Table 1:** **Baseline characteristics of included studies**

**Study**	***N*** **(P/Cntl)**	**Population**	**Age (P/Cntl)**	**Illness severity (P/Cntl) (mean)**	**Modality**	**Intensity**	**Replacement fluid**
Reeves and colleagues [23]	30 (14/16)	Sepsis syndrome	39/53 (mean)	26.2/24.2 (adults)	Plasma filtration	2 PV during first 4 to 6 hours, 3 PV throughout following 28 to 30 hours (total five volumes)	FFP/protein electrolyte replacement solution (1:4 ratio)
		22 adults					
		Eight children					
				26.3/26.9 (children^a^)	(PF1000 plasma filter, Gambro, Lund, Sweden)		
						213 ml/kg (SD 42.2) plasma exchanged	
				(APACHE II)			
Busund and colleagues [24]	106 (54/52)	Adults with severe sepsis or septic shock	41/48 (mean)	56.4/53.5	Plasmapheresis	One treatment of 30 to 40 ml/kg exchange	FFP:5% albumin replacement fluid (1:1 ratio)
				(APACHE III)	(PF-0.5 [Lvov, Russia] and DK2-03 [Rjazan, Russia] plasmapheresis machine)		
						(Repeated once if no clinical improvement)	
						Mean 1,820 ± 402 ml (first session) and 1,763 ± 312 ml (second session)	
Nguyen and colleagues [25]	10 (5/5)	Children with thrombocytopenia and multiorgan failure due to sepsis	1 to 16 /3 to 18 (range)	25.7/25.7	Plasma exchange (SPECTRA, Gambro BCT, Lakewood, Co, USA)	1.5 volumes day 1; 1.0 volumes for 14 days)	NR
				(PELOD)^a^			
						Median of 12 treatments given	
Long and colleagues [26]	48 (25/23)	Children with severe sepsis	2.8 (1.2 to 9.6) /2.8 (0.9 to 5)	NR	Plasma filtration	2 PV over first 2 hours (100 ml/kg) followed by 6 PV over the next 30 hours (300 ml/kg)	FFP/protein electrolyte replacement solution (1:4 ratio)
					(PF1000 or PF2000 plasma filter, Gambro, Lund, Sweden)		
			Median (IQR)				

APACHE, Acute Physiology and Chronic Health Evaluation; Ctrl, control; FFP, fresh frozen plasma; IQR, interquartile range; NR, not reported; P, plasmapheresis; PELOD, Pediatric Logistic Organ Dysfunction; PV, plasma volume; SD, standard deviation. ^a^Results inferred from published graph.

**Table 2:** **Risk of bias assessment of included studies**

**Study**	**Industry funding**	**Random sequence generation**	**Allocation concealment**	**Blinding (participants/personnel)**	**Blinding (outcome assessors)**	**Incomplete outcome data**	**Selective reporting**	**Other bias**	**Overall assessment**
Reeves and colleagues [23]	No	Low	Low	Unclear	Unclear	Low	Low	High^a^	High
Busund and colleagues [24]	NR	Unclear	Unclear	Unclear	Unclear	Low	Low	High^a^	High
Nguyen and colleagues [25]	No	Unclear	Unclear	Unclear	Unclear	Low	Low	Unclear	Unclear
Long and colleagues [26]	NR	Unclear	Low	Unclear	Unclear	Low	Low	High^a^	High

NR, not reported. ^a^High risk due to significant baseline imbalances.

### Primary outcomes and subgroup analyses

**Figure 2:**
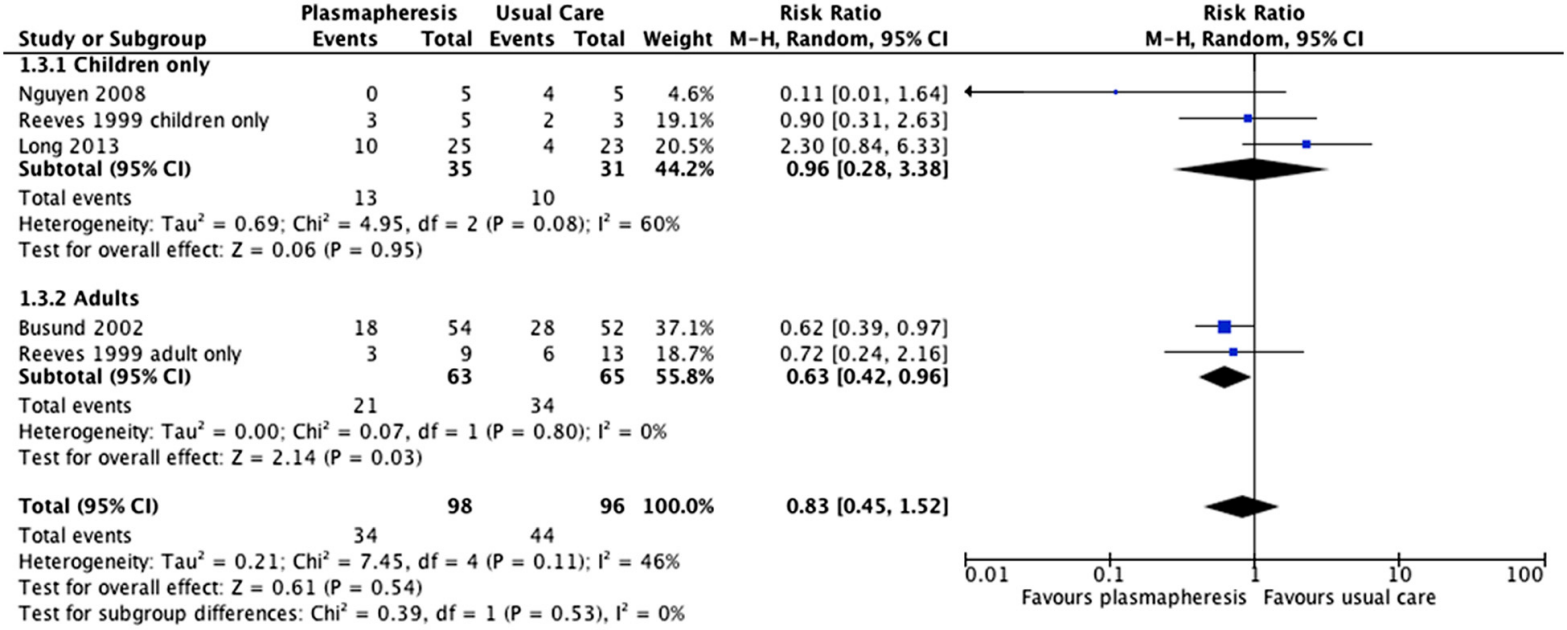
**Mortality associated with plasma exchange.** Boxes and horizontal lines represent point estimates, varying in size according to the weight in the analysis, and 95% confidence intervals. Chi^2^ = Chi-squared; df = degrees of freedom; CI = Confidence interval; I^2^ = I-squared; M-H = Mantel-Haenszel; P = P value; RR = risk ratio; Tau^2^ = Tau-squared; Z = Z score.

Mortality either at 14 days [23], at 28 days [24,26], or at an undefined time interval [25] was reported in the included trials. We pooled data from all four trials (*n* = 192) to generate a summary RR for mortality (Figure 2). Plasma exchange was not associated with a significant reduction in death from all causes (RR = 0.83, 95% CI = 0.45 to 1.52). The heterogeneity was moderate (*I*^2^ = 60%, uncertainly interval 0 to 87%).

To investigate the statistical heterogeneity, we performed subgroup analyses according to the age of the study population. In the two trials enrolling adult patients, the RR of mortality associated with plasma exchange was 0.63 (95% CI = 0.42 to 0.96, *I*^2^ = 0%, *n* = 128). In the pediatric population, the RR for death associated with plasma exchange was 0.96 (95% CI = 0.28 to 3.38, *I*^2^ = 60%, *n* = 66) (Figure 2). Subgroup analyses according to trial geography (for example, North American vs. other) resulted in nonsignificant and unstable point estimates (Additional file 4).

### Secondary outcomes

None of the included trials reported data on ICU or hospital lengths of stay. Similarly, no trials included data on central venous catheter-related complications. Only one trial reported adverse events associated with apheresis [24]. In this trial, six episodes of transient hypotension and one allergic reaction to fresh frozen plasma were reported in 81 plasma exchange procedures.

## Discussion

In this systematic review, we observed the overall RR for mortality in patients with sepsis treated with plasma exchange to be 0.83 (95% CI = 0.45 to 1.5, *I*^2^ = 60%). In adults, plasma exchange was associated with a significant reduction in all-cause mortality. Hospital and ICU lengths of stay and adverse events were insufficiently reported. Clinical heterogeneity among included trials was moderate and all trials were at unclear or high risk of bias.

The pathogenesis of sepsis involves systemic inflammation and upregulation of coagulation [27,28]. This response is characterized by the production of excessive inflammatory cytokines (including tumor necrosis factor alpha, interleukin-6, and interleukin-1β) [29], activation of coagulation and depletion of natural anticoagulants (antithrombin, tissue factor pathway inhibitor and protein C) [30,31]. Phase III trials examining replacement of deficient natural anticoagulants [32-35], and specific cytokines or circulating mediators [36-39] have shown inconsistent results. Therapies targeting broader aspects of immune homeostasis regulation of inflammation may be a more promising approach [5,40]. The role of plasma exchange has been well established in other conditions such as thrombotic thrombocytopenia purpura, a thrombotic microangiopathy associated with severely depleted levels of A Disintegrin-like and Metalloproteinase with Thrombospondin type-1 Motifs 13 (ADAMTS-13) [5,41]. Recently, ADAMTS-13 deficiency has been described in patients with severe sepsis and systemic inflammatory response syndrome, leading to an increased interest in the role of plasma exchange as an adjunct in the treatment of sepsis [25,42]. One included trial showed that ADAMTS-13 levels were significantly increased in patients randomized to receive plasma exchange [25]. Only one other trial provided data on inflammatory biomarkers, and this study showed significant decreases in plasma concentrations of C-reactive protein, alpha-1 antitrypsin, haptoglobin, and complement fraction C3 in patients who were randomized to plasma exchange [23].

Our meta-analysis builds upon a previously published review of blood purification modalities (including hemofiltration, hemoperfusion, plasma exchange or dialysis) in sepsis [43]. In that meta-analysis, authors restricted the analysis to adults and thus incorporated data from only two [23,24] of the four trials on plasma exchange included in our review. The diversity of blood purification methods included in the previous review is an additional limitation. To increase the homogeneity of the study intervention and generalizability of the meta-analysis, we included only trials that studied plasma exchange as the intervention.

Our search identified an additional trial examining a proprietary method of blood purification called coupled plasma filtration absorption, which combines, in series, a plasma filter, a nonspecific absorption column, and a hemodialysis filter [44]. Plasma was not replaced. This trial found that coupled plasma filtration absorption did not reduce mortality in patients with septic shock but the authors hypothesized that there may be a mortality reduction when high volumes of plasma were processed. We excluded this trial from the formal meta-analysis because the coupled plasma filtration absorption process involves renal replacement therapy as part of the intervention, which was an *a priori* exclusion criteria for our review.

The strengths of our systematic review are the comprehensive search strategy, which included multiple electronic database searches, hand searching of grey literature and bibliographies of included trials and forward searching. We used an *a priori* published protocol and followed the recommended guidelines for conducting and reporting systematic reviews. We performed rigorous assessments of bias of our included trials, which facilitated data interpretation in the context of trial methodology. Finally, we explored potential sources of clinical and statistical heterogeneity with *a priori* defined subgroup analyses.

There are several limitations of this systematic review that relate to the primary data. First, the small number of trials and patients enrolled is an important limitation of the data. Second, there were substantial baseline imbalances to consider in several of the included trials. Baseline differences such as younger age [23] and fewer patients requiring mechanical ventilation [24] in the intervention group may have biased the results of those included trials in favor of plasma exchange. In addition, one trial had higher illness severity scores in the plasma exchange group, which would have biased the mortality estimate in favor of the null hypothesis [26]. Technical differences in the application of plasma exchange existed among the included trials. The optimal plasma separation method, dose, and type of replacement therapy remain uncertain. These procedural details must be considered when deciding upon specific apheresis interventions (for example, dose, frequency, replacement fluid, duration of study, and so forth) in future trials.

Although all trials reported objective patient-oriented outcomes such as mortality, the duration of follow-up in these trials is short (14 or 28 days) and represents a further limitation of these data because follow-up of 90 days may be required to capture the full impact of interventions on longer term vital status in patients with sepsis [45].

An additional notable limitation is that only one trial reported adverse effects related to plasma exchange [24]. No trials addressed central venous catheter-related complications. Although thought to be a safe procedure, plasma exchange can be associated with cardiovascular instability, allergic reaction, or infection and/or thrombosis related to the presence of a large-bore central venous catheter [46,47].

## Conclusions

Insufficient evidence exists to recommend plasma exchange as an adjunctive therapy for patients with sepsis or septic shock. Rigorous randomized controlled trials evaluating clinically relevant patient-centered outcomes are required to evaluate the impact of plasma exchange in this condition.

## Key messages

- Sepsis and septic shock are leading causes of mortality in critically ill patients.
- Plasma exchange was not associated with a significant reduction in all-cause mortality overall, but was associated with a reduction in mortality in a subgroup of adult patients.
- Rigorous randomized controlled trials are required to evaluate the impact of plasma exchange in sepsis and septic shock.

## Additional files

Additional file 1: Table S1. Presenting the research question using the PICOS structure. Additional file 2: Table S2. Presenting the study eligibility criteria. Additional file 3: Table S3. Presenting the PubMed/MEDLINE search strategy. Additional file 4: Table S4. Presenting the mortality subgroup analyses.)

## Abbreviations

ADAMTS-13: A Disintegrin-like and Metalloproteinase with Thrombospondin type-1 Motifs 13
CI: confidence interval
RR: risk ratio
