# Supplementary material for: The efficacy and safety of plasma exchange in patients with sepsis and septic shock: a systematic review and meta-analysis
Source: Crit Care. 2014 Dec 20;18(6):699. doi: 10.1186/s13054-014-0699-2 (PMC4318234; doi:10.1186/s13054-014-0699-2)
Supplement: Additional file 1: Table S1. — Presenting the research question using the PICOS structure. [file 13054_2014_699_MOESM1_ESM.docx]

Additional file 1

Table S1 Research question using PICOS structure

| **Population** | Critically ill patients diagnosed with sepsis, severe sepsis, septic shock, or disseminated intravascular coagulation due to infection   - Both adults and children will be included |
| --- | --- |
| **Intervention** | Plasma exchange, or plasma filtration; regardless of timing, replacement fluid or frequency of administration |
| **Comparator** | Any comparator including placebo, standard of care, or no intervention |
| **Outcomes** | *Primary outcome:*   - All-cause mortality (longest reported follow-up)   *Secondary outcomes:*   - Hospital and ICU lengths of stay   *Safety outcome*:   - Central venous catheter-related complications (e.g. infection, thrombosis) |
| **Study design** | Prospective randomized controlled trials |
