# Supplementary material for: The efficacy and safety of plasma exchange in patients with sepsis and septic shock: a systematic review and meta-analysis
Source: Crit Care. 2014 Dec 20;18(6):699. doi: 10.1186/s13054-014-0699-2 (PMC4318234; doi:10.1186/s13054-014-0699-2)
Supplement: Additional file 2: Table S2. — Presenting the study eligibility criteria. [file 13054_2014_699_MOESM2_ESM.docx]

Additional file 2

Table S2 Study eligibility criteria

| **Inclusion Criteria:** | - Only prospective randomized trials will be considered - Patients (>80%) diagnosed with sepsis, severe sepsis, septic shock or disseminated intravascular coagulation (DIC) associated with infection - Plasma exchange, or plasma filtration; regardless of timing, number of treatments, replacement fluid or frequency of administration - Adults and children will be included |
| --- | --- |
| **Exclusion Criteria:** | - Observational study designs, quasi-randomized, crossover, or cluster-randomized trials will not be considered for this review - Studies involving animals - Studies with >20% of patients with disseminated intravascular coagulation due to causes other than infection (e.g. cancer-related DIC, obstetrical DIC) - Studies with no comparator group - Studies examining renal replacement therapy (including conventional dialysis, continuous renal replacement therapy, continuous veno-venous hemofiltration, or high-volume hemofiltration) |
