# Supplementary material for: The efficacy and safety of plasma exchange in patients with sepsis and septic shock: a systematic review and meta-analysis
Source: Crit Care. 2014 Dec 20;18(6):699. doi: 10.1186/s13054-014-0699-2 (PMC4318234; doi:10.1186/s13054-014-0699-2)
Supplement: Additional file 3: Table S3. — Presenting the PubMed/MEDLINE search strategy. [file 13054_2014_699_MOESM3_ESM.docx]

Additional file 3

Table S3 PubMed / MEDLINE search strategy

| 1. Sepsis[MeSH] 2. Sepsis 3. (Pyemia* OR Pyohemia* OR Pyaemia* OR Septicemia* OR (Blood AND Poisoning*) OR (Severe AND sepsis)) 4. Shock, septic[MeSH] 5. Shock, septic 6. (septic OR toxic OR endotoxic) AND shock 7. Disseminated intravascular coagulation[MeSH] 8. Disseminated intravascular coagulation 9. (Disseminated AND intravascular and coagulation*) OR (consumption AND coagulopath*) 10. Thrombocytopenia[MeSH] 11. Thrombocytopenia 12. #1 OR #2 OR #3 OR #4 OR #5 OR #6 OR #7 OR #8 OR #9 OR #10 OR #11 13. Plasmapheresis[MeSH] 14. Plasmapheresis 15. Plasma exchange[MeSH] 16. Plasma exchange 17. (Plasma[MeSH] OR Plasma) AND (Exchange[MeSH] OR Exchange) 18. Plasma Filtration 19. (plasma[MeSH] OR plasma) AND (filtration[MeSH] OR filtration) 20. Blood Component Removals[MeSH] 21. Blood Component Removals 22. (Blood AND Component AND Removal*) OR Pheres* OR Apheres* 23. #13 OR #14 OR #15 OR #16 OR #17 OR #18 OR #19 OR #20 OR #21 OR #22 24. (randomized controlled trial[pt] OR controlled clinical trial[pt] OR randomized[tiab] OR placebo[tiab] OR drug therapy[sh] OR randomly[tiab] OR trial[tiab] OR groups[tiab]) NOT (animals[mh] NOT humans[mh]) 25. #12 AND #23 AND #24 |
| --- |
