# Supplementary material for: The efficacy and safety of plasma exchange in patients with sepsis and septic shock: a systematic review and meta-analysis
Source: Crit Care. 2014 Dec 20;18(6):699. doi: 10.1186/s13054-014-0699-2 (PMC4318234; doi:10.1186/s13054-014-0699-2)
Supplement: Additional file 4: Table S4. — Presenting the mortality subgroup analyses.) [file 13054_2014_699_MOESM4_ESM.docx]

Additional file 4:

Table S4: Mortality subgroup analyses

| **Subgroup** | **Number of studies** | **Participants** | | **Effect measure**  **RR**  **(95% CI)** | **I^2^**  **(uCI)** |
| --- | --- | --- | --- | --- | --- |
|  |  | Plasma exchange (n/N) | Control  (n/N) |  |  |
| **Geography** |  |  |  |  |  |
| North America[[25](#_ENREF_25)] | 1 | 0/5 | 4/5 | 0.11  (0.01 to 1.64) | N/E |
| Other[[23](#_ENREF_23), [24](#_ENREF_24), [26](#_ENREF_26)] | 3 | 34/93 | 40/91 | 0.95  (0.47 to 1.91) | 64% |
|  | | | | | |
| **Risk of Bias** |  |  |  |  |  |
| High/Unclear[[23-26](#_ENREF_23)] | 4 | 34/98 | 44/96 | 0.84  (0.41 to 1.73) | 60% |
| Low | 0 | NE | NE | NE | NE |

RR = risk ratio; CI = confidence interval; uCI = uncertainty intervals; NE = not evaluable
